# Effective phase noise considerations in magnon based parametric excitations

**DOI:** 10.1038/s41598-021-90730-5

**Published:** 2021-05-31

**Authors:** Aneesh Venugopal, R. H. Victora

**Affiliations:** Department of Electrical and Computer Engineering, University of Minnesota Twin Cities, Minneapolis, 55455 USA

**Keywords:** Electrical and electronic engineering, Electronic and spintronic devices, Applied physics

## Abstract

Magnon-phase is an important entity in the parametric processes involving magnons, yet the general qualitative and quantitative consequences of the phase-noise on nonlinear properties remain far from understood. In the current simulation-based theoretical study, we explore the direct impact the phase-noise has on non-linearity. We use analytical techniques usually employed in the study of hydrodynamics to explain the magnon-based nonlinear phenomena. The behavior of the threshold-field and growth rate of the magnons in the presence of Gaussian phase-noise is analytically predicted. These predictions are verified by micromagnetic simulations. Such results are of crucial importance in the design and engineering of both traditional and futuristic devices.

## Introduction

The phenomenon of parametric resonance is one that occurs in diverse areas of physics such as hydrodynamics^1^, liquid crystals^2^, and electronic circuits^3^, to name a few. It involves the excitation of a natural mode of an oscillatory system using a spatial and/or temporal external agency. Modes in magnetic materials*—spin-waves,* or their quanta—*magnons,* allow for interesting parametric-resonance phenomena when excited by, for example, a microwave frequency. A quantity of great importance in parametric processes is the phase of the excited mode that plays an important role in producing interesting nonlinear phenomena.

Due to its direct involvement in nonlinear properties, the phase is also significant from a practical viewpoint, more so as the magnon-based devices become increasingly miniaturized. In fact, a myriad of applications that rely *directly* on the magnon phase have been proposed in the recent past. The magnon-based paradigm of applications—*magnonics*—has emerged as one of the promising candidates for information transfer and data-processing technology; with the magnon phase playing an important role in notable *novel applications* like spin wave based logic circuits^4,5^, reservoir computing & machine learning^6^, spin wave conduits for interconnects^7^, spin wave lens^8,9^, spin-based majority gate^10^, and other waveguide applications^11^. They are also valuable for spintronic applications. e.g., spin torque oscillators (STO)^12^ and neuromorphic computing^13^.

*Conventional applications* like telecommunication, satellite communication, and radar also use nonlinear magnetic devices to reduce energy consumption. Devices used in these applications, e.g., magnetics-based Frequency-Selective Limiters (FSLs), Signal to Noise ratio Enhancers (SNEs), phase shifters, etc.^14–17^ that rely on nonlinear absorption of microwave signals are invariably affected by the magnon phase. Often working in noisy radio-frequency (r.f) environments, these devices have to process/reject noisy signals impinging on them. However, magnon phase noise is rarely given any consideration in the design of such applications, and noise studies are often at the circuit level and phenomenological in approach^18^. However, as the demand for miniaturization grows, magnon phase-based nonlinear aspects and thereby the phase-noise itself have increasingly significant roles to play.

Phase noise has multiple sources; while the traditional magnet-based r.f devices that operate in high-noise environments are likely to encounter temporal phase-noise, newer magnonics applications employing smaller volumes of magnetic materials are more likely to be affected by process variations that result in spatial phase-noise. Effective spatial phase-noise in parametric processes may be attributed collectively to the presence of inhomogeneities, local impurities^19,20^, domains^21–24^, etc. In addition, the process of generation of the input microwave/spin-polarized current and the process of amplification of spin-waves can add temporal phase-noise into the magnetic system. Apart from the external sources, the distribution of k-vectors that is unavoidably present owing to the nonlinear nature of microwave excitation, can lead to potential decoherence of spin waves intrinsically^25^. However, this is usually neglected in nonlinear studies^26,27^, and often, assumptions of a single dominant mode are made.

Despite the importance of the magnon phase, the physical effects of phase-noise remain relatively unexplored in the larger context of magnon-based magnetics. Studies of noise in the past are largely based on thermal-noise where phase noise is often considered only in terms of macro-spin models and as a by-product of thermal-noise in the context of specific applications, e.g., STO^28–34^. A direct study of the phase-noise is, hence, timely. In this work, we present a more general and fundamental treatment of the consequences of phase noise, which is applicable across multiple magnon-based nonlinear applications. We verify the analytic conclusions from our studies with GPU-based micromagnetic simulations that allow the phase to be directly accessed, manipulated, and analyzed.

## Analytical theory and results

### Magnon growth

**Figure 1:**
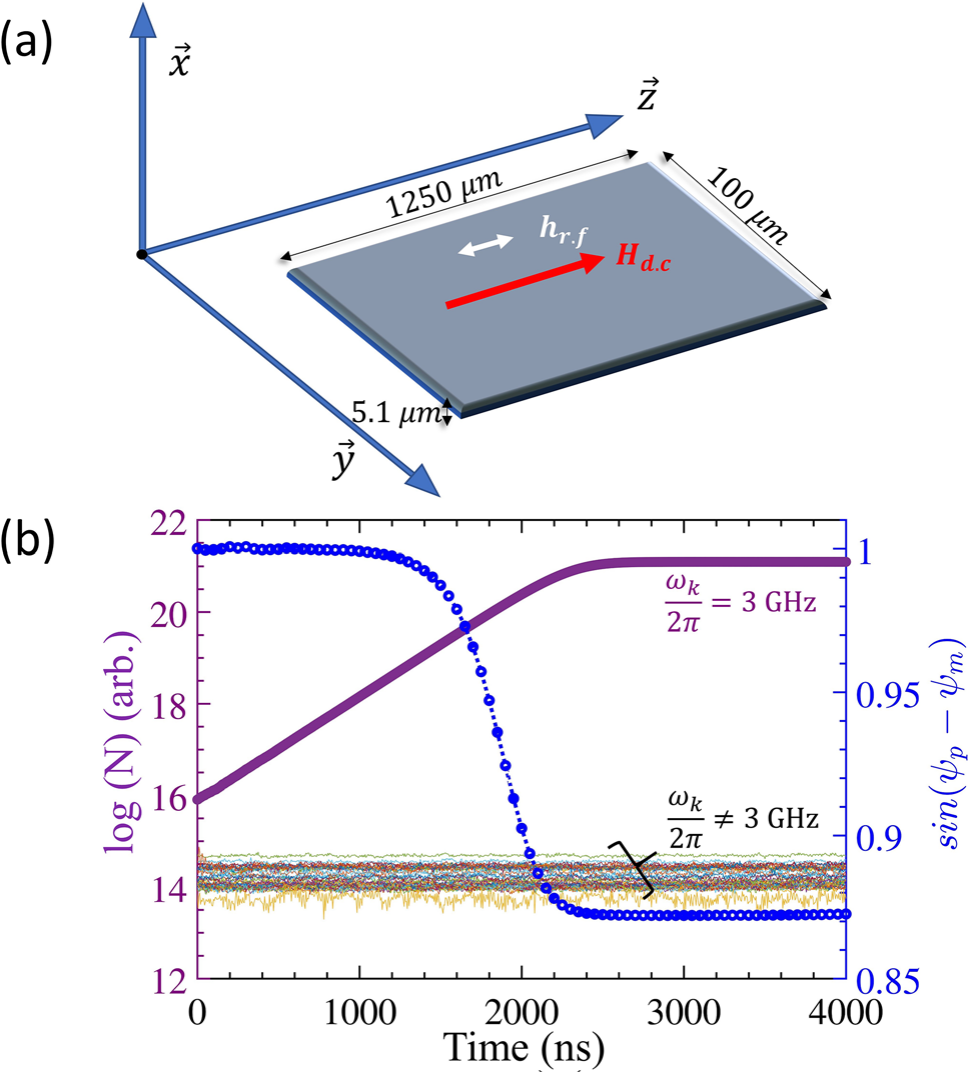
(**a**) Schematic of the sample and pump configuration used in the simulations. The microwave (r.f) signal, ${h}_{r.f}$ is applied parallel to the magnetic bias field, ${H}_{d.c}$. (**b**) Number of magnons, N, resolved in frequency for a pump-frequency of 6 GHz at 2.8 *Oe* and a bias-field of 200 *Oe*. ${\psi }_{p}$ and ${\psi }_{m}$ are the phases of the microwave pump and the dominant magnon mode, respectively. An exponential rise in numbers occurs only for magnons with frequency, $\frac{{\omega }_{k}}{2\pi }=3\:\mathrm{GHz}$ i.e., half the pump frequency, while the other magnons with $\frac{{\omega }_{k}}{2\pi }\ne 3\: \mathrm{GHz}$ remain close to their thermal equilibrium numbers.

We make use of the parallel-pump based microwave-excitation configuration wherein both the DC magnetic field that is used to bias the magnetic material and the microwave pump signal, $${h}_{r.f}=h\:cos({\omega }_{p}t)$$ ($$h$$ is the microwave field intensity and $${\omega }_{p}$$ is its angular pump frequency), are applied parallel to each other. The set-up schematic is shown in Fig. 1a. When the strength of the microwave field intensity exceeds a certain threshold ($$h={h}_{th}),$$ nonlinear processes are initiated in the magnetic material wherein magnons are excited due to the absorption of photons. In our case, it is the three-particle scattering process that leads to the growth of the magnons. In this process, a photon is absorbed and two magnons with half the photon energy are created while conserving the wave-vectors of the particles involved. If $${\omega }_{p}$$, $${\omega }_{{k}_{1}}$$, $${\omega }_{{k}_{2}}$$ represent the energies of the pump photon and the two excited magnons (with wave-vectors $${k}_{1}$$ and $${k}_{2}$$) respectively, then the following holds: $${\omega }_{p}={\omega }_{{k}_{1}}+{\omega }_{{k}_{2}}$$ such that $${\omega }_{{k}_{1}}={\omega }_{{k}_{2}}={\omega }_{p}/2$$ with $${k}_{1}+{k}_{2}$$ =0. This is the phenomena of parametric excitation of magnons at half the pump (or microwave) energy via the 3-particle scattering process, wherein the magnons are selectively excited. More details on such nonlinear processes can be found in our earlier works^35,36^. To understand the magnon growth process analytically, we start with the Landau-Lifshitz equation:

1 $$\frac{d\mathbf{M}}{dt}=-\gamma \left[\mathbf{M}\times \mathbf{H}\right]$$

where **H** is the effective magnetic field, and **M** is the magnetization vector. We can write, from the previous studies^35^, the equation of motion for magnetization in terms of magnon operators:

2 $$\frac{d{b}_{k}}{dt}-i{\omega }_{k}{b}_{k}=-ih{V}_{k}{b}_{-k}^{*}\mathrm{cos}\left({\omega }_{p}t\right)$$

${V}_{k}$ is the coupling factor between the microwave and the dominant magnon mode; ${b}_{-k}^{*}$, ${b}_{k}$ represent the conjugate creation-annihilation pair of magnon operators. Without loss of generality, we assume a solution of the form ${b}_{k}\sim {e}^{i{\varpi }_{k}t}$. (Note, when $h=0$, ${b}_{k}\sim {e}^{i{\omega }_{k}t}$.) The forcing term, $h\:\mathrm{cos}\left({\omega }_{p}t\right)$, can initiate resonance when ${\varpi }_{k}=-{\varpi }_{k}\pm {\omega }_{p}$ implying $\left|{\varpi }_{k}\right|={\omega }_{p}/2$, which is not unexpected due to the nature of 3-particle interaction. Note that Eq. (1) ignores the phenomenological damping that is usually added to the Landau-Lifshitz equation to account for the losses. We introduce to the microwave-phase a Gaussian temporal phase-noise, $\stackrel{\sim }{\phi }$ of zero-mean and standard deviation, $\sigma$:

3 $$\frac{d{b}_{k}}{dt}=i{\omega }_{k}{b}_{k}-ih{V}_{k}{b}_{-k}^{*}\mathrm{cos}\left({\omega }_{p}t+\stackrel{\sim }{\phi }\right)$$

### Method of scales

Next, we use the method of scales^20^ to gain insights into the equation of dynamics [Eq. (3)]. We start by making the following definitions: $\delta \equiv |{\omega }_{k}-\frac{{\omega }_{p}}{2}|$ such that, $\delta \equiv \epsilon\Delta$ and $T\equiv \epsilon t$, where $\delta$ represents the detuning of the dominant spin wave frequency from half the pump-frequency and *T* represents a smaller time-scale that would be useful in obtaining the amplitude equation of the magnon mode; $\epsilon$ represents a small parameter $(\epsilon \ll 1)$. Also, under normal pumping conditions, $h{V}_{k}\ll$ ${\omega }_{k}, {\omega }_{p}$ so that we can define $h{V}_{k}$ $\equiv \epsilon {\omega }_{p}F$, such that *F* is proportional to the input excitation.

In view of the above definitions, we have $\frac{d}{dt}=\frac{\partial }{\partial t}+\epsilon \frac{\partial }{\partial T}.$ We make an ansatz that ${b}_{k}$ has the following perturbation expansion:

4 $${b}_{k}={u}_{0}\left(t,T\right)+{\epsilon u}_{1}\left(t,T\right)+\dots$$

wherein, ${u}_{1}$, can be interpreted as a higher harmonic correction to the principal term, ${u}_{0}$. We can write up to order ${\epsilon }^{1}$:

5 $$\frac{d{b}_{k}}{dt}=\frac{\partial {u}_{0}}{\partial t}+\epsilon \left(\frac{\partial {u}_{0}}{\partial T}+\frac{\partial {u}_{1}}{\partial t}\right)$$

The right-hand side of Eq. (3), evaluates to:

6 $$\frac{d{b}_{k}}{dt}=i\frac{{\omega }_{p}}{2}{u}_{0}+ \epsilon \left(i\frac{{\omega }_{p}}{2}{u}_{1}+i\Delta {u}_{0}-i{\omega }_{p}F{u}_{0}^{*}\mathrm{cos}\left({\omega }_{p}t+\stackrel{\sim }{\phi }\right)\right)+O({\epsilon }^{2})$$

Using Eqs. (5) and (6) above, the equation of ${u}_{0}$: $\frac{\partial {u}_{0}}{\partial t}-i\frac{{\omega }_{p}}{2}{u}_{0}=0$ or $\mathcal{L}.{u}_{0}=0$ where $\mathcal{L}\equiv \left(\frac{\partial }{\partial t}-i\frac{{\omega }_{p}}{2}\right)$, has a plane-wave solution:

7 $${u}_{0}\left(t,T\right)=A(T){e}^{i\frac{{\omega }_{p}}{2}t}$$

with *A(T)* being the complex amplitude of ${u}_{0}$. At the next order ${\epsilon }^{1}$, we have

8 $$\mathcal{L}.{u}_{1}=-\frac{\partial {u}_{0}}{\partial t}+i\Delta {u}_{0}-i{\omega }_{p} F{u}_{0}^{*}\mathrm{cos}\left({\omega }_{p}t+\stackrel{\sim }{\phi }\right)$$

Using (7) above, we have:

9 $$\mathcal{L}.{u}_{1}=\left[-\frac{dA\left(T\right)}{dT}+i\mathrm{\Delta A}\left(\mathrm{T}\right)- i\frac{{\omega }_{p}}{2}F{A}^{*}\left(T\right)<{e}^{\stackrel{\sim }{\phi }}>\right]{e}^{i\frac{{\omega }_{p}}{2}t}-\left[- i\frac{{\omega }_{p}}{2}F{A}^{*}\left(T\right)<{e}^{\stackrel{\sim }{\phi }}>\right]{e}^{-i\frac{{3\omega }_{p}}{2}t}$$

Note that ${e}^{i\frac{{\omega }_{p}}{2}t}$ is a solution of the homogeneous equation: $\mathcal{L}.{u}_{1}=0.$ Therefore, if in (9), ${e}^{i\frac{{\omega }_{p}}{2}t}$ has non-zero coefficients, it would lead to solutions of ${u}_{1}\left(t,T\right)$ that are secular in *t*, which would eventually diverge, leading to ${u}_{1}$ exceeding ${u}_{0}$. We avoid the secularity by suppressing the resonant term^37^, i.e., by requiring that $A\left(T\right)$ satisfies the following:

10 $$-\frac{dA\left(T\right)}{dT}+i\mathrm{\Delta A}\left(\mathrm{T}\right)- i\frac{{\omega }_{p}}{2}F{A}^{*}\left(T\right)<{e}^{\stackrel{\sim }{\phi }}>=0$$

Finally, using the property of Gaussian functions $<{e}^{\stackrel{\sim }{\phi }}> = {e}^{-\frac{{\sigma }^{2}}{2}}$, we have the amplitude equation to be:

11 $$\frac{dA\left(T\right)}{dT}=(\Gamma +i\Delta )\mathrm{A}\left(\mathrm{T}\right)- i\frac{{\omega }_{p}}{2}F{A}^{*}\left(T\right){e}^{- \frac{{\sigma }^{2}}{2}}$$

Since $$h\propto F,$$ this implies $${h}_{thn}\propto {h}_{th0}{e}^{\frac{{\sigma }^{2}}{2}}$$, where $${h}_{th0}$$ and $${h}_{thn}$$ represent the threshold field intensities in the absence and presence of phase-noise, respectively. In Eq. (11), $$\Gamma$$ is a phenomenological loss parameter. Hence, as far as the nonlinear behavior is concerned, in the presence of a temporal Gaussian phase-noise, the threshold-field depends exponentially on the noise variance. Another important result is the dependence of the slope of the growth rate ($${g}_{r}$$) w.r.t the microwave intensity ($$h)$$. It is deducible using Eq. (11) that $$\frac{{dg}_{rn}}{dh}={\frac{{dg}_{r0}}{dh}e}^{-\frac{{\sigma }^{2}}{2}}$$. While the threshold is an important parameter, especially for conventional applications, as discussed earlier, the growth rate plays a crucial role in the transient-state dynamics.

## Simulation methods

To test the above results, we next perform micromagnetic calculations. We simulate a thin film of the magnetic insulator YIG using saturation magnetization, ${M}_{S}=145\: {\mathrm{emu}/\mathrm{cm}}^{3}$, intrinsic damping constant, $\alpha =0.0007$, exchange constant, ${A}_{ex}=3.77\times {10}^{-7}\frac{\mathrm{ergs}}{\mathrm{cm}}$ and gyromagnetic ratio, $\gamma =1.76\times {10}^{7}\frac{\mathrm{rad}}{\mathrm{s} \mathrm{Oe}}$. The sample measures $5.1$ µm (thickness; along x) $\times 100$ µm (along y)$\times 1250$ µm (along z) and periodic boundary conditions are used. The sample is discretized into $128\times 128\times 1$ cells for the simulations. Note that we use a higher discretization in the x–y plane which helps us conserve computational resources and runtime while enabling high-resolution studies in the plane into which the dominant magnons scatter^35,36,41^. Thermal effects at 300 K are taken into account using an effective magnetic field. The demagnetization field is implemented as per Newell et al.^38^.

The Landau–Lifshitz–Gilbert equation for the cells is solved using a CUDA-based micromagnetics solver employing graphics processing units (GPU)^35,39,40^. A fixed bias field of ${H}_{d.c}=200$ *Oe* is chosen to saturate the magnetic material. A microwave pump frequency of $\frac{{\omega }_{p}}{2\pi }=6\: \mathrm{GHz}$, which constitutes an off-resonant pumping with respect to the chosen bias-field ($\frac{{\omega }_{res}}{2\pi }=1.78 \: \mathrm{GHz}$ at 200 *Oe*), is used to excite the magnons.

## Discussion

The simulation conditions are such that the 3-magnon scattering dominates (also called the first-order Suhl process^26^). Figure 1b illustrates the exponential growth of 3 GHz magnons for pumping slightly above the microwave threshold-field for a pump frequency of 6 GHz and a bias-field of 200 Oe. This growth eventually saturates due to the phase-mismatch between the photons and the magnons. The phase-match, $\mathrm{sin}\left({\psi }_{p}-{\psi }_{m}\right),$ between the dominant magnon phase, ${\psi }_{m}$, and the pump microwave phase, ${\psi }_{p}$, controls the energy transferred from the microwave to the magnons. However, 4-magnon interactions amongst the exponentially growing 3 GHz magnons can cause $\mathrm{sin}\left({\psi }_{p}-{\psi }_{m}\right)$ to differ from 1, reducing the coupling and eventually resulting in magnon growth saturation^41^. As the pump phase is deterministic and constant, it is the magnon-phase that plays a key role in this process. The phase-mismatch mechanism is intrinsic; nevertheless, it points to the important role of the magnon phase in governing the nonlinear behavior of the magnetic system. As mentioned earlier, the simulation results in Fig. 1b show the selective excitation of the ${\omega }_{k}/2\pi =3 \:\mathrm{GHz}$ mode while the rest of the modes (thermally generated and shown as a colored band in Fig. 1b) with ${\omega }_{k}/2\pi \ne 3\: \mathrm{GHz}$ hardly shown any increase.

**Figure 2:**
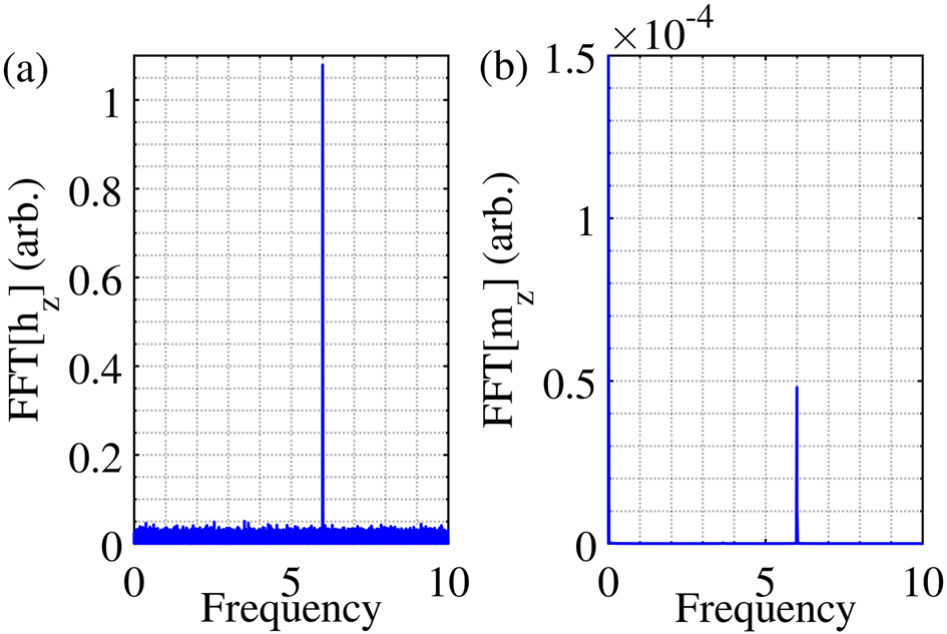
Selective property of parametric pumping. When a 6 GHz microwave signal is employed in the presence of phase-noise with zero mean and standard deviation of 80°, there occurs noise-rejection at frequencies other than 6 GHz in the absorption. (**a**), (**b**) represent the Fourier transform of the microwave input signal and the longitudinal component of magnetization (${m}_{z}$) respectively. A microwave field of 7.5 Oe is used.

As discussed earlier, however, the phase mismatch can also be extrinsically induced by phase-noise, $$\phi \left(t\right)$$. In line with the theory developed in the previous section, we study the effects of Gaussian phase noise on the magnetic system. Compared to the mean, it is the standard deviation, $$\sigma ,$$ of the phase-noise, $$\phi \left(t\right),$$ that has a dominant impact on the nonlinear behavior of the system; this has been verified in the simulations as well. Consequently, we set the mean of the phase-noise to zero and vary the $$\sigma$$. In Fig. 2, we plot the Fourier transform of the spatially averaged longitudinal component, $${m}_{z}$$, and that of the noisy microwave signal with a mean of 0 and $$\sigma =80^\circ$$. It can be observed, as indicated earlier, that the selective nature of parametric resonance largely amplifies only the 6 GHz component of $${m}_{z}$$. (Note that $${m}_{z}$$ oscillates at twice the frequency of $${m}_{y}$$ (or $${m}_{x})$$ ^27^) It is observed, however, that the threshold-field increased from 2.7 *Oe* in the absence of phase-noise to 6.6 *Oe* in its presence.

**Figure 3:**
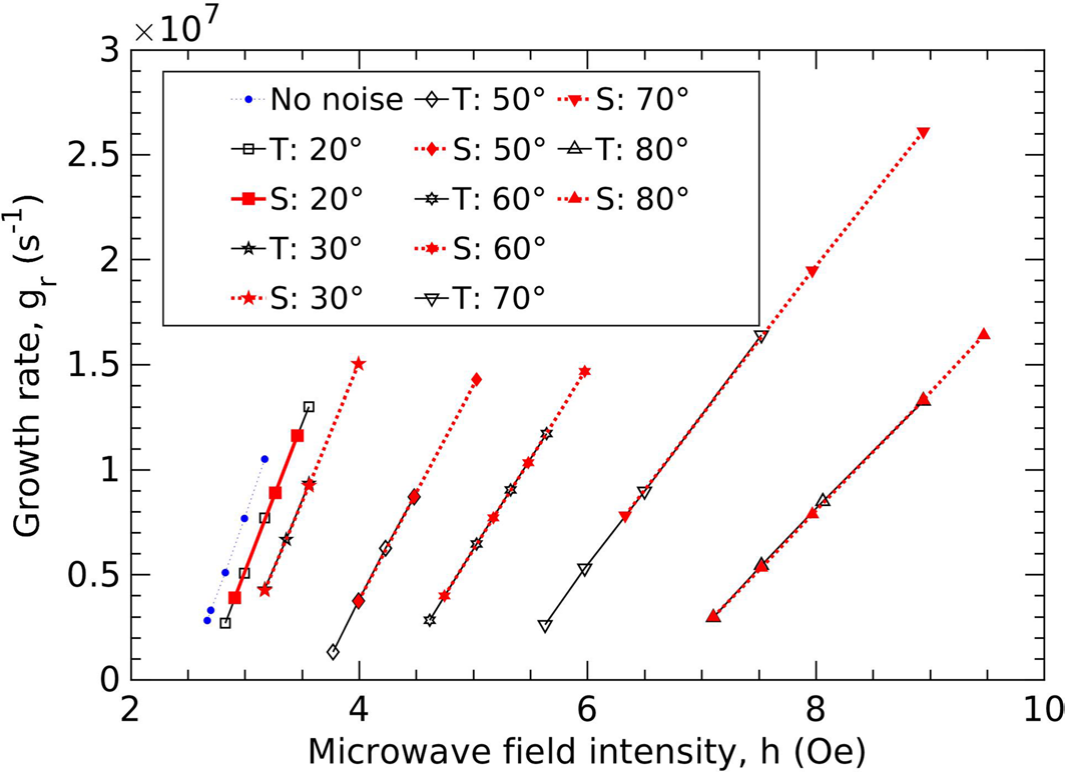
Growth rate of magnons vs. the primary r.f field intensity for various phase-noise standard deviations. ‘T’ refers to temporal noise, ‘S’ to spatial noise. The black solid lines with hollow symbols correspond to temporal noise while the red dotted lines with filled symbols correspond to spatial noise. The quantity in degrees indicates the standard deviation, $\sigma$, of the phase noise.

The modified threshold-fields can be obtained from the x-intercepts of the growth-rate (of the magnons) versus the microwave-field plots, as shown in Fig. 3. The growth rate for the various simulation conditions can, in turn, be determined from the slope of the logarithmic plots of the magnon number against time, e.g., using data for t < 2500 ns in Fig. 1b. (Beyond a standard deviation of ~$100^\circ$ for the phase noise, the threshold field becomes unreasonably high.) Physically, one can see this behavior to be a consequence of the direct relationship between the phases of the external excitation agency and the magnons. As mentioned earlier, threshold-field plays an important role in governing the nonlinear behavior of the magnetic material^11,17,42,43^.

**Figure 4:**
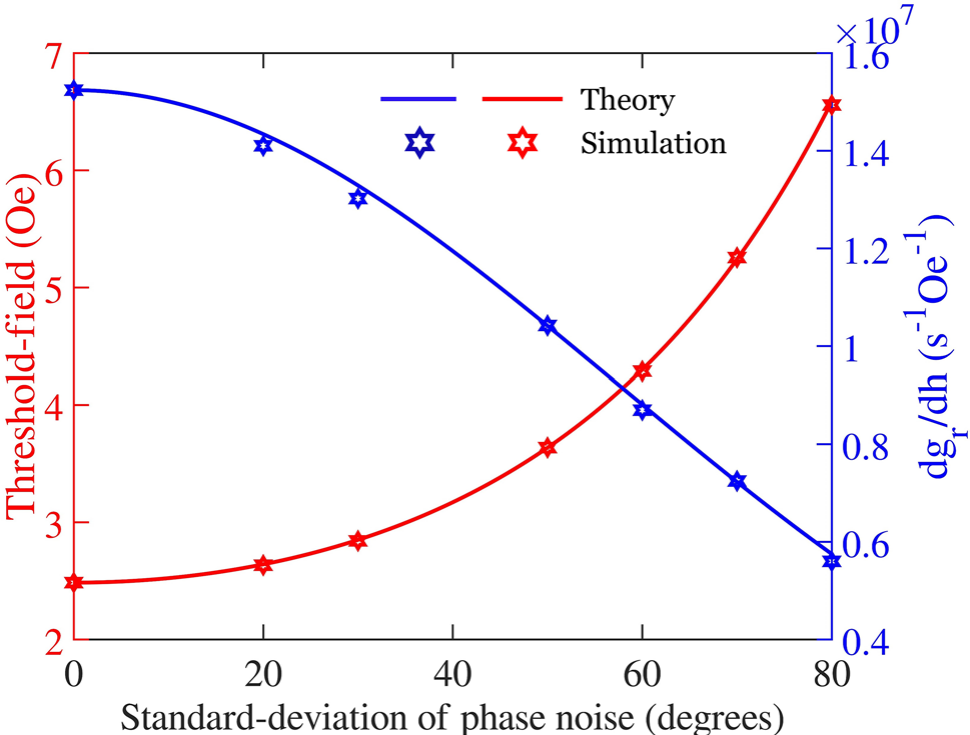
Comparison of simulated data and theoretical predictions of threshold-fields and the slope of growth rate, $\frac{d{g}_{r}}{dh}$ w.r.t microwave field intensity.

The other important inference from Fig. 3 is the reduction in the slope of the growth rate ($\frac{{dg}_{rn}}{dh}$) of the magnons with an increase in $\sigma$, as was also deduced from the analytical equations earlier. This is proportional to the coupling factor, ${V}_{k}$ [using Eq. (2)] and thereby determines the energy coupling from the microwave into the magnetic material. The threshold field increment and the decrement in the slope of the growth rate as obtained from the simulation and theory are shown in Fig. 4. The growth rate is a crucial quantity involved in the determination of the response time of the magnetic system. It constitutes part of the transient state duration before the magnon growth is limited by higher-order processes (phase-mismatch mechanism), as shown in Fig. 1b. $\frac{{dg}_{rn}}{dh}$ and hence, ${V}_{k}$ plays an especially important role in the mechanics of ferromagnet-based microwave cavity experiments^44–46^. The impact of the phase noise on $\frac{{dg}_{rn}}{dh}$ is non-trivial, since, in our earlier parametric studies, performed in the presence of an additional secondary frequency^36^ we did not observe such an effect, although we did predict and demonstrate an increase in the threshold field. These results are important for both the traditional and novel classes of applications discussed earlier since device performance characteristics often strongly depend on threshold-field as well as the transient-time. This is evident, e.g., in the ongoing efforts to miniaturize nonlinear magnetic rf device—FSL, especially for autonomous automobile applications^15^.

We also computed, using our simulation system, the shift in the threshold field in the presence of Gaussian *spatial* phase noise $\phi (x)$, where $x$ represents the spatial location $x$ in the magnetic sample. Mathematically, we can consider the effective microwave-field at the location $x$: $h\left(x\right)=h\: \mathrm{cos}\left({\omega }_{p}t+\phi \left(x\right)\right)$. The simulation results are nearly identical to the curves obtained using the temporal noise (Fig. 3). As before, the mean of spatial phase noise is kept zero in all the cases. Hence, it is interesting that the threshold-field shift and the slope-reduction effect caused by the spatial noise turn out to be nearly identical to that in the case of temporal noise. This points to the importance of process variations and microstructural quality in the production of magnetic samples.

## Conclusion

To summarize this work: a new way of understanding the consequences of phase noise is presented. The methodology described helps us to gain new insights into the behavior of magnons under phase-noise. This is independent of the mode of pumping (parallel or perpendicular) and the magnetic-material (applies to insulators, e.g., YIG as well as metals, e.g., NiFe). In fact, the results in general could be used to understand the nonlinear properties of other parametric systems that involve three-particle processes.

It is observed that both the threshold-field intensity and the growth rate of magnons scale exponentially with the variance of the effective phase-noise in the system. Moreover, both the spatial and temporal variance can contribute to the changes in the nonlinear properties. It should be stated that noise need not necessarily be a nuisance, but it can make possible system behavior that does not exist in the absence of noise. For example, from the results, it can be noted that the threshold-field can be deterministically increased by adding phase-noise into the magnetic system. Clearly, a proper understanding of the consequences of magnon phase-noise is not just interesting from a physical perspective for revealing new phenomena, but also crucial from an application perspective in order to elicit desired functionalities in magnon-based devices.
